# Affective concordance in couples: a cross-sectional analysis of depression and anxiety consultations within a population of 13,507 couples in primary care

**DOI:** 10.1186/s12888-017-1354-7

**Published:** 2017-05-19

**Authors:** J. Walker, J. Liddle, K. P. Jordan, P. Campbell

**Affiliations:** 1 School of Medicine, Faculty of Medicine and Health Sciences, Keele University, Keele, Staffordshire UK; 2 Arthritis Research UK Primary Care Centre, Institute for Primary Care and Health Sciences, Keele University, Keele, ST5 5BG UK; 3 Institute of Health and Society, Newcastle University, Newcastle upon Tyne, UK

**Keywords:** Anxiety, Depression, Primary care, Concordance, Mental health, Family, Couples, Consultation

## Abstract

**Background:**

Depression and anxiety are common and have a significant impact on the individual and wider society. One theory proposed to explain a heightened risk for depression and anxiety is affective concordance in couples (e.g. influence of shared mood states, shared health beliefs). Whilst research has shown concordance for severe psychiatric illnesses and general mood in couples, little attention has been given to concordance for common psychiatric conditions such as depression and anxiety. The aims of this study were to test affective concordance in couples and examine potential influences on concordance.

**Methods:**

Study design is a 1-year cross-sectional study of anxiety and depression consultations in primary care. Data were obtained from a validated primary care database of recorded consultations. Outcome was the presence of an anxiety or depression Read Code (GP recorded reason for consultation) in the female (within the couple dyad), and exposure was a recorded Read Code of anxiety or depression in the male. Logistic regression was used to test associations with odds ratios (OR) and 95% confidence intervals (95% CI) reported. Statistical adjustment was carried out on potential influences of concordance; age, environment (deprivation), healthcare behaviour (consultation frequency), and comorbidity.

**Results:**

A population of 13,507 couples were identified in which 927 people consulted for anxiety and 538 for depression. Logistic regression showed a 3 times increase in odds of an anxiety consultation in females if their male partner had also consulted OR 2.98 (95% CI 2.15 to 4.13). For depression females were over 4 times the odds of consulting if their male partner had also consulted OR 4.45 (95% CI 2.79 to 7.09). Adjustment within a multivariable model showed some reduction in odds; concordant anxiety was reduced to 2.5 times odds OR 2.48 (95%CI 1.76 to 3.50) and depression reduced to OR 3.39 (2.07 to 5.54).

**Conclusion:**

Results show significant associations for affective concordance in couples. Factors influencing concordance are comorbidity and environmental factors, however reasons for deciding to consult (positive or negative) are unknown. This study highlights the patients’ social context as a factor in consultations for anxiety and depression and gives support to the consideration of the patient’s household as an influence on mental health.

## Background

The global burden of disease study in 2010 showed that mental health and substance use disorders are the leading cause of years lost to disability worldwide [1]. Anxiety and mood disorders, most prominently depression, are the most common forms of mental disorders, and collectively pose an increasing challenge for healthcare, with an estimated global cost of US $16 trillion projected for the next 25 years in the U.S.A [2]. A similar picture exists within the UK, where the National Audit Office states that NHS England spent an estimated £11.7 billion on mental health services in 2014–15, which made up 12% of total NHS spending [3]. Depression and anxiety are a major priority for healthcare, not only because of their direct effect on the individual, but also their significant presence in the risk and prognosis of most chronic conditions that impact society [4]. Prevalence of depression and anxiety is high: life time estimates of clinical level depression and clinical level anxiety are approximately 15% [5, 6], with sub clinical threshold levels much higher [7–9].

There are a number of factors associated with risk of depressive and anxiety disorders, such as gender, presence of comorbidity or other chronic disease, genetics and gene/environment interactions, negative life events (current and past), coping, self-efficacy, deprivation, education, social support, previous history, and social participation [10–14]. One notable social phenomenon is shared concordance or shared risk in family members and couples (e.g. families, marital partners, partners that live together). Research has shown associations between family members and couples in a range of health conditions; coronary heart disease [15], hypertension [16], hyperlipidaemia [17], lung cancer [18], diabetes mellitus [19], musculoskeletal health [20], and mental health [21, 22]. Genetic (between related family members), behavioural, and environmental influences have been proposed to explain such concordance within families and couples [15]. Couples (i.e. married couple, partners living together) who live together are subjected to similar behavioural and environmental factors which may increase risk of disease concordance [23]. These shared behavioural and environment influences can relate to external factors that the couple may have little control over, such as shared financial hardship, shared deprivation, or shared bereavement [24]. It may also include lifestyle-orientated factors that couples may have more control over, such as a shared diet, shared health risk behaviour (e.g. smoking, alcohol intake), shared physical activity levels, or direct effects within the relationship such as marital discord [25–28]. There are also positive reasons for concordance within a consultation population, it may be that one partner has received beneficial treatment for depression or anxiety and this encourages the other partner to seek healthcare.

One important component of concordance is affective concordance, which refers to shared emotional states in partners. Goodman and Shippy [29] describe the relationship between partners as “one of interdependence and reciprocity”, where factors that influence emotion in one partner can influence the mental state of the other. This assimilation process between partners can encourage positive health behaviours, however such affective influences could also be risk factors for depression or anxiety [23]. For example Joutsenniemi et al. [30] report significant concordance for severe psychiatric disorders within couples in a large population sample; and a paper by Hippisley-Cox et al. [17] demonstrated concordance for depression within couples in a primary care sample in the UK. However, authors of a recent meta-analysis of the link between couples and health related outcomes have stated the mechanisms of how and why such effects occur are largely unknown [31]. In a more specific review of affective concordance research, the authors state that previous studies lack consistency in methods, measures of affect are often low quality, samples are small, and there is a lack of theoretical testing [15]. For example, the Hippisley-Cox et al. [17] study on couples within primary care reported on concordance for depression, however they did not adjust for potentially important confounders such as consultation frequency (i.e. was concordance explained by a higher propensity for couples to consult overall rather than for depression?), or assess any other theoretical influences (e.g. shared deprivation, shared comorbidity) that may explain the effects that were reported. More information is required to understand concordance in couples, to firstly enable a better understanding of what components influence concordance, and secondly to highlight which of these may be targets for future interventions.

## Methods

### Aim

This study aims to establish whether concordance for depression and anxiety is present within couples in a primary care consultation sample. The study will also examine potential theoretical influences on concordance, such as age, comorbidity, shared deprivation, and shared health behaviour.

### Setting

This was a cross-sectional study of primary care health consultation records over a 1-year period (1st January 2006 to 31st December 2006). Consultation records are appropriate for research given that over 97% of the UK population are registered with a general practice [32]. Health consultations were identified through the Consultations in Primary Care Archive (CiPCA), an anonymised medical record database which collates patient data from 13 North Staffordshire GP practices [33]. Participating practices have been trained and assessed in the quality of their morbidity recording [34], and CiPCA has been shown to have comparability in consultation prevalence to other UK national primary care medical record databases [33]. CiPCA has ethics approval from North Staffordshire and Staffordshire Research Ethics Committees. Electronic health records such as CiPCA are established in health research and have been used to investigate disease epidemiology, comorbidity, prognosis, process and delivery of care, and evaluate outcomes of care in a wide spectrum of diseases and illnesses [35, 36].

### Participants and procedure

In this study, 13,507 couples registered at their local GP practices were included in the analysis. Using previous methodology to identify couples within electronic health record databases [17, 20] the following criteria were applied; couples both being aged 30–74 years, having the same address, being of different genders, having an age difference of no more than 15 years, and having no other adult aged 30–74 within the household. These criteria have been shown to be valid [17] and reduce the chance of including adult/parent child dyads, but is limited as it excludes same sex couples and couples where one or more additional adult is present within the household and may include adult siblings of opposite sex. Within the analysis the male within the couple dyad was assigned as the exposure partner, and the female partner as the outcome partner, this is an arbitrary choice but follows previous methodology [17, 20]. Exposure was defined as a recorded Read code indicating a depression or anxiety consultation in the male partner, with the outcome determined as a recorded Read code for the depression or anxiety in the female partner within the same 12-month period. General practitioners within the UK enter medical diagnosis or symptoms using Read codes which are organised into a hierarchical recording system. Consultations included presenting to a GP at a practice, a home visit by GP or a telephone consultation that resulted in the recording of a diagnostic Read code or Symptom code for depression or anxiety. Multiple consultations on the same day were recorded as ‘one’ contact.

### Exposure and outcome

**Fig. 1:**
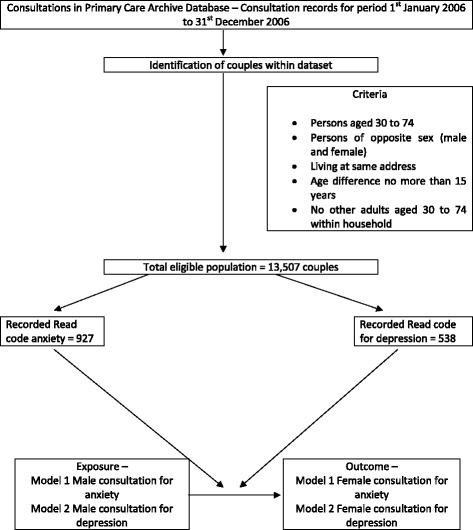
Flow diagram of recruitment process

The exposure and outcomes for this study were a recorded consultation Read code for depression (depressive disorders, dysthymia, mood state) or anxiety (anxiety disorders, panic disorders) within the male (exposure) and female (outcome) of the couple dyad. The study tested the association between depression as exposure and depression as outcome, and anxiety as exposure and anxiety as outcome in two separate models. The Read Code System was used to identify relevant mental health consultations (NHS Information Authority, 2000) using the UKTC (UK Terminology Centre) [37]. Read codes for mental health conditions such as depression and anxiety, used within general practice within the UK have been developed to map with ICD 10 chapter v coding (Classification of Mental and Behavioural Disorders) [38]. Relevant Read codes for anxiety and depression used in this study were selected from chapter E “Mental Disorders” of the UKTC following previous methodology [20, 39], these include codes for; anxiety, anxiety disorder, phobic anxiety, social phobic disorders, generalised anxiety disorder, low mood, depressive symptoms, depressed mood, dysthymia, reactive depression, major depressive episode, recurrent depressive disorder, as well as many more (all codes used are available on request). Please refer to Fig. 1 for a flow diagram of the recruitment and analysis process.

### Potential influences on concordance

A number of proxy measures were taken to assess potential influences on the level of affective concordance between couples based on previous research which has shown such influences on depression and anxiety outcomes [40–42]. We included a measure of the presence of a cardiovascular disease consultation, as previous research (see review, [15]) has demonstrated concordance in couples, and explanations for this concordance encompass broader shared lifestyle factors (e.g. diet, exercise, general health). Read Codes relating to hypertension, ischaemic heart disease, cerebrovascular disease, hypotension, dyslipidaemia, were included following previous methodology [39]. This study also included the indication of musculoskeletal condition consultation as research has shown concordance in couples [20], and musculoskeletal conditions are associated with increased disability, burden, and psychological distress including anxiety and depression [43]. Read Codes from chapter N were included to indicate a musculoskeletal condition consultation in either partner following previous methodology [20]. A further two potential influencing variables were created to account for the effects of psychiatric comorbidity; for example the presence of depression may influence an anxiety consultation and vice versa, as both conditions are often correlated within primary care populations [44]. The variables (termed here “psychiatric comorbidity”) are; i) where anxiety is the outcome; a variable was determined whether either or both of the couple dyad had a depression consultation, ii) where depression is the outcome; a variable was created to indicate either or both of the couple dyad had a consultation for anxiety. For shared healthcare engagement (i.e. healthcare use) the frequency of consultations (regardless of reasons for consultation) were used. Categories of consultation frequency were created with those (male or female) in the top 20% of frequency categorised as “frequent consulters” following previous methodology [20, 45]. To control for shared deprivation, the neighbourhood deprivation status of couples was determined using the UK Index of Multiple Deprivation (IMD, Office for National Statistics, [46]). The deprivation variable was separated into 3 categories to indicate the 20% most and least deprived, and the middle 60% deprived, following previous methodology [20, 47]. To determine the effect of age on health concordance, the patients were grouped into age bands (30–39 years, 40–49 years, 50–59 years, 60–69 years, 70+ years, [48]).

### Statistical analysis

Odds ratios (OR) and 95% confidence intervals (95% CI) were calculated using logistic regression. Two analyses were performed, the first was the examination of the association between anxiety consultations of male partners (exposure) with anxiety consultations in their female partners (outcome), and for the second, exposure and outcome were consultations for depression. Three stages were carried out within each analysis. Stage 1: unadjusted association between exposure and outcome. Stage 2: several models that examined each potential theoretical influence (shared comorbidity, shared healthcare engagement, shared deprivation, and age) on the unadjusted associations found in stage 1 by adding each influence variable in separate models. Stage 3: multivariable adjustment; all aforementioned potential influences of health concordance were included simultaneously to determine their combined effects on the relationship between exposure and outcome. Due to collinearity between female and male partner age (*r* > 0.9), only female age banding was used within these analyses. Further exploratory analysis was carried out on the potential influencing variables (musculoskeletal consultation, shared psychiatric comorbidity, shared healthcare engagement, deprivation) that independently reduced the association between the exposure (male partner consultation for anxiety or depression) and outcome (female consultation for anxiety or depression) to assess their independent effects by male or female partner as well as in combination. Variables were created for this analysis to show female consultation for these influence variables and their effect on outcome, male consultation for these influence variables and their effect on outcome, and where both within the couple dyad had consulted, using percentage proportions and odds ratio with 95% confidence intervals. Finally sensitivity analysis was carried out whereby the male (exposure) and female (outcome) multivariable model was reversed (i.e. female consultation is now exposure, male consultation as outcome) to determine if differences exist dependent on couple assignment within the model (data not shown).

## Results

Total eligible population was 27,014 individuals, equating to 13,507 partner dyads. The mean age was 52 years (female = 51, male = 53), and the mean number of all consultations was 5 (median 3) within the 12-month study period. Overall there were 927 (3.4%) patients recorded with an anxiety consultation, and 538 (2.0%) patients recorded with a depression consultation. Females consulted more than twice as much for anxiety conditions (4.7%) as males (2.2%); a similar pattern was found for depression consultations (2.8% females, 1.2% males). 17 (0.1%) males had consulted for both depression and anxiety, and 38 (0.3%) females had consulted for both. Table 1 outlines the characteristics of the cohort.

**Table 1:** Participant characteristics

	Mean	95% CI	Median	IQR
Age				
Male	53	52.8–53.2	53	44–62
Female	51	50.9–51.3	51	42–60
Consultation frequency over 12-month period	4.9	4.9–5.0	3	1–6
Males	4.2	4.15–4.31	3	1–6
Females	5.6	5.54–5.72	4	2–8
Consultation prevalence	Males	Females	Both partners	
Anxiety consultation prevalence	292 (2.2%)	635 (4.7%)	44 (0.3%)	
Depression consultation prevalence	163 (1.2%)	375 (2.8%)	21 (0.2%)	

*IQR* inter quartile range, *CI* 95% confidence interval

Unadjusted logistic regression results (Table 2) show that females had significantly increased odds of an anxiety consultation if their partner had a recorded anxiety consultation (OR 2.98, 95% CI 2.15, 4.13). A similar significant association was found for depression; with females whose partner had consulted for depression having over four times the odds of also having a depression consultation (OR 4.45, 95% CI 2.79, 7.09), compared to females whose partner had not consulted.

**Table 2:** Unadjusted associations of concordance for anxiety and depression consultations between partners

	Male with consultation		Male without consultation		Odds ratio	95% Confidence interval
Condition	*n*	Female with consultation *n* (%)	*n*	Female with consultation *n* (%)		
Anxiety	336	44 (13.1%)	13,171	635 (4.8%)	2.98	2.15, 4.13
Depression	184	21 (11.4%)	13,323	375 (2.8%)	4.45	2.79, 7.09

There was no impact on these estimates after adjustment for cardiovascular comorbidity (Table 3), however small reductions in the strengths of association were identified after adjustment for musculoskeletal consultations and for psychiatric comorbidity. Adjustment for healthcare engagement (consultation frequency) in the model gave the greatest reduction in odds for both anxiety and depression. Adjusting for deprivation had no effect on the association between males and female consulting for anxiety, but did show a reduction in odds for depression. Adjustment for age did not markedly alter the association for either anxiety or depression. After adjustment for all factors, the odds ratio for the outcome of anxiety reduced from 2.98 (unadjusted model) to 2.48 (95% CI 1.76, 3.50). The final depression multivariable model showed a more marked reduction after adjustment for all factors, OR reducing from 4.45 (unadjusted model) to 3.39 (95% CI 2.07, 5.54) in the adjusted model.

**Table 3:** Multivariable adjusted models for concordance for anxiety and depression consultations between partners

			Adjusted models: Odds Ratio (95% Confidence Interval)					
	Unadjusted	Comorbidity			Shared healthcare engagement	Shared deprivation	Participant age (females)	Final multivariable model
		CVD	MSK	Psychiatric				
Anxiety	2.98 (2.15, 4.13)	2.94 (2.12, 4.08)	2.85 (2.06, 3.96)	2.88 (2.08, 4.01)	2.61 (1.86, 3.67)	2.96 (2.13, 4.11)	2.96 (2.14, 4.11)	2.48 (1.76, 3.50)
Depression	4.45 (2.79, 7.09)	4.51 (2.83, 7.19)	4.29 (2.69, 6.85)	4.20 (2.63, 6.72)	3.83 (2.36, 6.23)	4.28 (2.69, 6.83)	4.41 (2.76, 7.04)	3.39 (2.07, 5.54)

*CVD* cardiovascular disease, *MSK* musculoskeletal

Table 4 shows the factors from the adjusted model that independently reduced the association between exposure and outcome (female consultation for anxiety or depression). Overall the main significant effects shown are for female presence of musculoskeletal consultations, psychiatric comorbidity consultations, and frequency of consultations. Additional presence of these in males did not markedly strengthen these associations. Increasing deprivation had a significant association with female depression consultation but not with anxiety.

**Table 4:** Influence of musculoskeletal consultations, shared healthcare engagement, shared psychiatric morbidity and deprivation on female anxiety and depression consultations

Outcome	Variable	Influence	Percentage females consulted for outcome	OR (95% CI)
Anxiety	Musculoskeletal consultation	No partner consulted	4.0%	Reference
		Male partner consulted	5.2%	1.31 (1.06, 1.63)
		Female partner consulted	6.3%	1.59 (1.31, 1.92)
		Both partners consulted	6.7%	1.69 (1.33, 2.15)
	Shared psychiatric comorbidity^a^	No partner consulted	4.9%	Reference
		Male partner consulted	7.4%	1.56 (0.86, 2.82)
		Female partner consulted	9.6%	2.08 (1.46, 2.96)
		Both partners consulted	9.5%	2.06 (0.48, 8.87)
	Shared healthcare engagement	No frequent consulters	3.3%	Reference
		Male frequent consulter	4.3%	1.31 (1.03, 1.67)
		Female frequent consulter	9.9%	3.18 (2.63, 3.84)
		Both frequent consulters	11.8%	3.86 (3.05, 4.88)
	Shared deprivation	Low deprivation	4.7%	Reference
		Mid deprivation	5.0%	1.08 (0.88, 1.32)
		High deprivation	5.5%	1.18 (0.93, 1.51)
Depression	Musculoskeletal consultation	No partner consulted	2.6%	Reference
		Male partner consulted	2.3%	0.89 (0.65, 1.20)
		Female partner consulted	3.5%	1.36 (1.06, 1.75)
		Both partners consulted	4.7%	1.86 (1.40, 2.48)
	Shared psychiatric comorbidity^a^	No partner consulted	2.7%	Reference
		Male partner consulted	5.8%	2.21 (1.34, 3.65)
		Female partner consulted	5.8%	2.21 (1.56, 3.14)
		Both partners consulted	2.3%	0.83 (0.11, 6.06)
	Shared healthcare engagement	No frequent consulter	1.7%	Reference
		Male frequent consulter	2.2%	1.34 (0.95, 1.87)
		Female frequent consulter	7.2%	4.58 (3.61, 5.81)
		Both frequent consulters	7.1%	4.52 (3.34, 6.12)
	Shared deprivation	Low deprivation	2.2%	Reference
		Mid deprivation	2.9%	1.32 (0.99, 1.75)
		High deprivation	3.7%	1.71 (1.24, 2.35)

OR (95% CI), Odds Ratio (95% Confidence Interval)

^a^Presence of depression consultation in partners when outcome is anxiety in female, presence of anxiety consultation in partners when outcome is depression in female

Results of the sensitivity analysis whereby exposure and outcome were reversed (i.e. female partner consultation as exposure, male partner consultation as outcome) showed no marked difference from the model used in this study; anxiety final multivariable model (OR 2.46 95% CI 1.74, 3.47), depression final multivariable model (OR 3.39 95% CI 2.07, 5.55).

## Discussion

Female partners are more likely to have a consultation for anxiety or depression if their male partner has also consulted for the same condition. These effects are partially explained by the presence of comorbidity, healthcare engagement, and deprivation. These findings support the affective concordance hypothesis of shared mental health state in couples, and highlight the potential contextual influences on the rates of consultations for depression and anxiety in primary care.

### Comparison with previous literature

Previous literature has highlighted the presence of health concordance in couples and families [15, 49], and this current study reports expected associations for the presence of affective concordance in couples within a primary care consultation sample. A study by Nilsen et al. [50], using a multilevel model for anxiety and depression outcomes in couples, found a couple clustering effect with 19% of variance explained at the couple rather than individual level for anxiety, and 25% variance explained for depression, suggesting a potentially stronger effect for depression, which is reflective of the results reported in this study. A more directly comparable study by Hippisley-Cox et al. [20], which used a similar primary care medical record database and Read codes, showed a higher prevalence of depression overall (males 6%, females 13%), however their study included Read codes for prescriptions and treatments and this may have inflated overall prevalence.The results reported by Hippisley-Cox et al. for depression show an odds ratio (OR 2.18, 95% CI 1.8, 2.7) compared to this study’s stronger effect (OR 3.39). This difference may be explained by including prescriptions and treatments in the definition of depression within the Hippisley-Cox study, which may have led to a greater case mix overall. A longitudinal cohort study by Joutsenniemi et al. [30] considered the relationship between psychiatric disorders in one partner and the development of psychiatric disorders in the other, and report significant concordance association effects. They adjusted their results for age, sociodemographic factors (education, household income, deprivation), and relationship factors (partner age difference, children in household) and report some reduction in their effects due to adjustment, similar to the effects of adjustment found in this current study. Overall this current study supports the literature that affective concordance is present between couples, and has now demonstrated this phenomena exists for depression and anxiety consultations within a primary care population, as well as examined potential influences for concordance. However it needs to be noted that this study considers health seeking behaviour (i.e. consultations) and the drivers for this may also include witnessing positive benefits of treatment within a partner which has motivated someone to also seek healthcare.

### Strengths and weaknesses

One of the major strengths of this study is the large sample size, representative of a general population sample of couples aged between 30 and 74, given that over 97% of the UK population are registered with a primary care GP [32]. Another strength is the use of a primary care database; such databases have proved reliable, ethical and suitable for epidemiological studies [50, 51], and are not subject to selection and recall bias associated with questionnaire based designs [52]. A further strength of this study is the examination of potential influences on concordance, something called for within the literature [31] and not fully addressed in previous studies. A key statistical adjustment in this current study is the influence of consultation frequency in both partners, as this illustrates that affective concordance is not a consequence of frequency of visits to the general practitioner. Though adjustment for this variable within the regression model reduced the magnitude of concordance, and further exploratory analysis showed independent effects from the individual and from the partner on prevalence of anxiety and depression consultations based on consultation frequency. This study also examined other important potential influences that may represent aspects of shared health and lifestyle (comorbidity), and shared environment (deprivation). Though effects from these variables are minimal within each individual adjusted analysis (e.g. Table 4), the combined effects show some explanation for concordance overall, suggesting aspects of shared lifestyle and environment exert some influence on affective concordance in couples.

Whilst attempts were made to account for potential shared influences within the analysis, the measures of these influences are limited within medical record data. The CiPCA dataset, as with many other primary care medical record datasets, is restricted on the information about the individuals’ lifestyle (e.g. alcohol intake, diet, physical activity). These factors along with health behaviours and beliefs are not routinely recorded by GPs [53, 54]. Deprivation scores were linked to neighbourhoods and no information could be given on individual aspects of deprivation (employment status, household income, education and skills, overcrowding, access to amenities), such information may have given a more accurate indication of influence within the analysis. The use of specific Read codes that indicate cardiovascular disease may give some insight into the shared health and lifestyle between couples, however they are not in themselves indicative of the shared health behaviour and lifestyle of couples. Similarly the use of Read codes for musculoskeletal consultations were used to give an indication of comorbidity and potentially shared disability which can impact on depression and anxiety, however more information would be required on actual impact of a musculoskeletal condition (e.g. severity, interference, disability). There was also no information on the quality of the relationship within the partnership, whether marital discord is a contributor to depression and anxiety states within couples, or on family structure and parenthood status (e.g. if couple have children and the age of children). Research has shown the influence of discord at both a partner and family level and the link to depression and anxiety outcomes, with prospective evidence that marital discord precedes affective states [28, 55]. Due to the cross sectional design of this study there is no indication of causality (i.e. which partner consulted first), and there is no information on the duration of anxiety or depression, as a consultation does not necessarily signify the beginning of an episode. Furthermore we have no information on the actual severity of anxiety or depression in either exposure or outcome partner, or what reasons partners choose to consult (i.e. other factors aside from anxiety or depression that may have influenced consultation). There is no information on couples below the age of 30 years and over the age of 74 or same sex couples where associations may have differed. Finally not everyone with anxiety and depressive symptoms will consult, and indeed partners may actually influence the reason not to consult. Prospective designs are needed within the primary care population to help ascertain how concordance develops over time between couples.

### Clinical relevance

Results show the association between one partner’s consultation for anxiety or depression and the other partner’s consultation for the same condition. The magnitude of this effect is notable with a two and a half increase in odds for an anxiety consultation, and a three and a half increase in odds for a depression consultation. This indicates social contextual factors that may be important to consider when patients present and consult for anxiety or depression within primary care. Certainly such influences between partners and families are not new within the literature in terms of more severe psychiatric conditions (schizophrenia, bi-polar disorder, major depressive disorder). A wealth of research has demonstrated the effect of the family/home environment on recovery and illness management, notably the work on expressed emotion [56, 57]. Studies on expressed emotion have shown that addressing family environment influences through increased education and understanding about the condition, and improved communication between family members, can have a significant impact on relapse rates [56, 57], and this may be an approach that could be adopted for less severe mental health conditions. Aside from this potential partner’s “reaction or expressed emotion” to the other partner’s state as a reason for concordance (i.e. increase psychosocial stress in reaction to partner’s anxiety/depression), there may be other negative shared life events (e.g. death in the family) that may contribute to concordance, and it may be beneficial for clinicians to ask about the impact of such events at a partner or family level to give greater perspective on the context of the consultation. Another important consideration is the level of relationship quality and marital discord present, depression and anxiety may well be a symptom of such discord [55], and may signify the need to access relationship counselling or couple therapy. Of course there may well have been more positive reasons for concordance. It may be equally true that partners have consulted about their mental health because they have witnessed successful treatment, and positive benefits from treatment, within their partner and have decided to consult themselves. However the conclusions outlined above necessitate the need for further longitudinal research to ascertain the developmental and dynamics aspects of affective concordance in couples.

Another perspective for clinical relevance is at the public health level as the results, if extrapolated to a population level are large (i.e. 3 to 4 times the odds of consultation) and indicate the potential for taking a family level view on treatment. Studies have shown that family/partner level interventions on modifiable lifestyle factors for people affected by diabetes and coronary heart disease can reduce the impact of those conditions [58–60]. The results of this study do show some attenuation of odds, when accounting for deprivation, musculoskeletal health, and psychiatric morbidity suggesting potential targets to increase mental well-being in couples and perhaps families. There is also the evidence of independent effects both within the individual and the partner associated with higher female anxiety and depression consultation prevalence (Table 4). For example a small increase in estimated prevalence (4.0 to 5.2%) for anxiety consultation in female partners if their male partner has a musculoskeletal condition, or if the male is a frequent consulter (3.3 to 4.3%). It may be that such effects lead to collective stress between partners (e.g. family level poverty, caring for someone with a musculoskeletal disability or a mental health condition). Perhaps consideration could be given to wider contextual influences that may increase the likelihood for partner or family level consultation for mental health conditions within primary care and from that suitable interventions directed at couples/families be developed. Again further work is now required to understand these potentially partner/family level effects on consultation.

## Conclusion

In conclusion this study has demonstrated an increase in the likelihood of an anxiety or depression consultation if a partner also consulted for the same consultation. Potential influences on this concordance are comorbidity and some shared environmental factors. This study highlights the patients’ social context as a base for understanding consultations for anxiety and depression and gives support to the consideration of the patient’s household as an influence on patient’s mental health.

## Abbreviations

CiPCA: Consultations in Primary Care Archive
GP: General Practitioner
ICD: International classification of disease
IMD: Index of multiple deprivation
NHS: National Health Service
UKTC: UK Terminology Centre
